# The Longitudinal Interplay between Adverse Peer Experiences and Self-Regulation Facets: A Prospective Analysis during Middle Childhood

**DOI:** 10.1007/s10802-023-01117-1

**Published:** 2023-10-02

**Authors:** Nele Westermann, Robert Busching, Annette M. Klein, Petra Warschburger

**Affiliations:** 1 Department of Psychology, University of Potsdam, Karl-Liebknecht-Straße 24/25, 14476 Potsdam, Germany; 2 International Psychoanalytic University Berlin, Stromstr. 1, 10555 Berlin, Germany

**Keywords:** Self-regulation, Executive function, Victimization, Rejection, Middle childhood, Longitudinal

## Abstract

Positive peer experiences and self-regulation (SR) skills are crucial for children's healthy development, but little is known about how they interact during middle childhood. Therefore, we examined the prospective links between adverse peer experiences (APEs) and SR, drawing from the dataset of the PIER study. Across three measurement points, 1654 children aged 6–11 (T1), 7–11 (T2), and 9–13 years (T3) were included. We assessed the SR facets updating, flexibility, inhibition, emotional reactivity, inhibitory control, and planning using computerized tasks, parent- and teacher-reports. The latent variable of APEs consisted of measures of peer victimization and peer rejection assessed via self-, parent-, and teacher-report. Separate cross-lagged panel models were calculated, investigating the interplay of each SR facet and APEs. Results indicated that experiencing more APEs at T1 predicted higher emotional reactivity, and lower inhibition, inhibitory control, updating, and flexibility at T2. More APEs at T2 predicted higher emotional reactivity and lower planning at T3. Lower inhibition, updating, and flexibility at T2 predicted more APEs at T3. Accordingly, we found a negative bidirectional relationship between inhibition, updating, and flexibility with APEs. Our findings highlight that during middle childhood more APEs predict lower SR, which in turn predicts more experiences of peer victimization and rejection.

**Supplementary Information:**

The online version contains supplementary material available at 10.1007/s10802-023-01117-1.

## Introduction

Middle childhood encompasses the age range between 6–12 years and is characterized by new developmental tasks demanding shifts in cognitive, emotional, and social behavior (DelGiudice, 2018). Important contributors to development during middle childhood include the extent of self-regulation (SR) and the quality of peer relationships (Poulin & Chan, 2010). Current research suggests a negative association between problems with peers and SR (Pratt et al., 2014; Robson et al., 2020). However, in the sense of the chicken-or-egg dilemma, uncertainty about the temporal relationship between these two factors exists. Do problems with peers predict lower SR capacity or vice versa? Understanding this interplay is essential when looking from a preventive and intervention-specific perspective.

### Adverse Peer Experiences in Childhood

As children enter school, they spend more and more time with their peers, and peer relationships become increasingly important and challenging (Lam et al., 2014). Consequently, not only positive experiences with peers occur. Data from the PISA study 2018 (OECD, 2019) indicated that across OECD countries, an average of 8% of children reported being excluded and bullied, and another 14% reported that other children made fun of them at least a few times a month. Adverse peer experiences (APEs) include experiences of victimization and rejection by peers. Peer victimization is the experience of physical or psychological harm caused by peers acting outside of the norms of appropriate conduct (Finkelhor et al., 2012). In contrast, peer rejection is defined as the group's dislike of an individual (Lopez & DuBois, 2005). Even though peer victimization and rejection are operationalized differently, they share the perpetrators' intent to cause harm and an imbalance of power between perpetrator and victim (Graham & Bellmore, 2007). APEs were found to lead to adverse developmental outcomes such as aggression (Herts et al., 2012), and anxiety and depression (Adrian et al., 2019). Interestingly, internalizing symptoms and aggression were also found to be predictors of peer victimization (Brendgen et al., 2016). When examining peer rejection and externalizing problems in childhood bidirectionally, empirical evidence supported both directions, in the sense that externalizing problems predicted peer rejection which in turn predicted more externalizing problems (Sturaro et al., 2011). It is therefore essential to consider and investigate both directions—the underlying mechanisms leading to APEs as well as the potential effects of APEs. One interindividual variable that needs to be discussed in both directions is SR.

### Self-Regulation

A recent meta-analytic review by Robson et al. (2020) emphasized the critical role of SR in childhood development, as SR levels predicted interpersonal behavior, mental health, and achievements later in life. SR is considered a broad psychological construct which we define as the ability to control behavior, cognition, emotion, and physiological reactions to attain future benefits (Baumeister & Vohs, 2004; Hofmann et al., 2012). SR encompasses different facets that become more distinct, differentiated, and interconnected throughout development (Nigg, 2017). Furthermore, a hierarchical structure is assumed in which basal SR facets enable more complex SR facets (McClelland et al., 2018). The executive functions (EF) working memory updating (updating), cognitive flexibility (flexibility), and inhibition can be considered as basal facets of SR (Gettens & Gorin, 2017; Warschburger et al., 2023) and should be considered in SR research (Bailey & Jones, 2019; Hofmann et al., 2012). Updating describes the ability to monitor and update working memory representations; flexibility is the ability to shift between mental sets or tasks, and inhibition is the ability to suppress primary behavioral impulses (Diamond, 2013; Miyake et al., 2000). Another basal facet of SR is emotional reactivity, which refers to a child's emotional reaction regarding the response threshold, latency, amplitude, intensity, and recovery time (Rothbart & Derryberry, 1981). In contrast to other SR facets, a higher level of emotional reactivity is considered to reflect a lower level of SR. Planning is an example of a complex SR facet that describes the ability to cope with tasks that need to be mastered currently or in the future (Roth et al., 2014). To date, there is no scientific consensus on a unifying theory of SR (Bailey & Jones, 2019), and numerous constructs are grouped together under the umbrella term of SR (Burman et al., 2015). Notably, studies suggested that individual SR facets are not highly intercorrelated (Duckworth & Kern, 2011; Eisenberg et al., 2019) and mature at different rates (Nigg, 2017). Therefore, differential developmental patterns are expected and it is important to examine the SR facets individually.

### The Link between Adverse Peer Experiences and Self-Regulation

In their meta-analytical review, Robson et al. (2020) indicated that children with a mean age of 7.5 years were less likely to be victimized if they had higher SR levels. An additional meta-analysis by Pratt et al. (2014) confirmed that lower SR was associated with more victimization in children and adults. In summary, meta-analytical studies pointed towards a negative association between APEs and SR. However, a pending question concerns the direction of this effect.

On the one hand, higher SR could make children less likely to be victimized or rejected by peers. Research indicated that successful peer interactions require friendly and self-regulated behaviors and actions (Hay et al., 2004). SR enables individuals to adapt to social situations and inhibit maladaptive impulses and is essential for socially appropriate behavior (Beauchamp & Anderson, 2010). Therefore, it is unsurprising that research has shown that children with more effortful and behavioral control are more likely to be popular (Maszk et al., 1999; Walker et al., 2001). In a 6-month follow-up study, 9- to 13-year-old children with dysregulated emotional reactivity had an increased risk of experiencing peer victimization (Rosen et al., 2012). A further study by Verlinden et al. (2014) assessed parent-reported inhibitory control, flexibility, emotional reactivity, updating and planning at the age of 4, and bullying involvement in the first years of elementary school. Results indicated that children who were victims of bullying showed lower inhibitory control levels. Thus, preliminary findings suggest low SR as a risk factor for experiencing APEs in childhood.

On the other hand, negative peer experiences could lead to lower SR. Even though SR is often considered genetically predisposed, evidence found that it is sensitive to life influences (Diamond & Lee, 2011). In this regard, negative experiences with peers can be interpreted as a stressor with negative implications for SR and its development (Blair, 2010). Indeed, a series of experimental studies manipulated social exclusion through fake feedback stating that the subject ends up alone in life and observed a depletion of SR capabilities in young adults (Baumeister et al., 2005). Farley and Kim-Spoon (2014) argue that adolescents with unsupportive peer relationships might have problems developing SR abilities, as good peer relations may act as a context for practicing and enhancing SR. Accordingly, APEs could represent a social context both influenced by SR and also influencing SR.

Theoretical considerations and first evidence are thus available for both directions, indicating the importance of directly investigating a potential bidirectional association between APEs and SR. In adolescence, a bidirectional relation between SR and positive peer relationships was found, such that better SR contributed to better peer relations and vice-versa (for a review, see Farley & Kim-Spoon, 2014). In early childhood, social exclusion at age 4 predicted decreased SR at age 6 and vice-versa (Stenseng et al., 2015). Few studies have examined the possible bidirectional relationships between APEs and SR in middle childhood. Lecce et al. (2020) assessed children aged 8 to 12 three times over one year. Their findings indicated that peer rejection negatively predicted inhibition but not updating. Lower scores of updating and inhibition were not related to later peer rejection assessed via peer nominations. An additional longitudinal study by Holmes et al. (2016) investigated the developmental association of APEs and SR from preschool (at age 4.5) to adolescence (at age 15). A composite EF score was formed by employing a multimethod, multi-informant approach, and APEs were assessed through reports from parents, children, and afterschool caregivers. Their results showed that APEs at age 6 predicted decreased EF in children aged 9 to 10. Additionally, lower EF at 4.5 years was associated with increased APEs in children aged 6 and 9 to 10. The previously highlighted findings suggest initial evidence for a possible negative bidirectional relationship between APEs and SR. In addition, this relationship may differ depending on age and SR facet.

### The Current Study

Previous literature suggests an association between APEs and SR in middle childhood, but the direction of this relationship remains unclear. Whether experiencing more APEs in middle childhood predicts individual SR facets or if individual SR differences predict the likelihood of experiencing more APEs has not been answered yet. Several shortcomings of previous research should be mentioned. First, prospective studies are rare despite being essential to investigate the direction of effects. Second, existing prospective studies either treated SR as a common factor (Holmes et al., 2016; Stenseng et al., 2015) or solely focused on single SR facets (Lecce et al., 2020). Finally, middle childhood, a period marked by many changes in both APEs and SR in the first years of school, has received little attention. Consequently, there is a call for further research to investigate the relationship between peer experiences and SR, focusing on a differentiated view of SR (Cumming et al., 2020).

The present study aimed to address these research gaps by investigating the bidirectional relations between different SR facets and APEs (peer victimization and peer rejection). As distinct SR facets, we included updating, flexibility, inhibition, emotional reactivity, inhibitory control, and planning. Using data from three waves in a longitudinal design, bidirectional relations between SR and APEs were investigated across middle childhood. The following hypotheses were tested: First, children with lower SR capacities at T1 or T2 were expected to experience more APEs at T2 and T3, respectively. Second, children experiencing more APEs at T1 or T2 were expected to indicate lower SR capacities at T2 and T3, respectively. We assumed negative predictive and concurrent associations between APEs and the SR facets inhibition, flexibility, updating, planning, and inhibitory control for both hypotheses. Emotional reactivity was expected to have positive predictive and concurrent associations with APEs.

Based on inconsistent findings regarding sex and age differences in the interplay between APEs and SR, potential sex and age differences were analyzed on an exploratory level. While most studies found no sex differences (Holmes et al., 2016; Robson et al., 2020), initial evidence suggested a stronger association of APEs and SR in boys compared to girls (Jenkins et al., 2018). Looking at age differences, Holmes et al. (2016) reported weaker associations with increasing age, supported by Pratt et al. (2014), who found marginally weaker self-control effects on victimization in adults compared to children. Age differences during middle childhood, however, need further exploration.

Preregistration increases transparency and reduces *p*-hacking and publication bias (Nosek et al., 2018; Simmons et al., 2021). Therefore, the study's hypotheses and analysis plan were preregistered; see https://osf.io/r6t3s. Minor deviations from preregistration are explained in the supplementary materials (Table S1).

## Method

### Procedure

Data were collected as part of a multifaceted prospective study investigating intrapersonal developmental risk factors in childhood and adolescence (PIER-study; Warschburger et al., 2023). The measurements took place in three waves in 2012 (T1), 2013 (T2), and 2015 (T3). Our study design covered middle childhood with different time intervals between T1 and T2 (about 9 months) and T2 and T3 (about 24 months) partly for organizational reasons, as the children were assessed during school hours. Participants were recruited from 33 public primary schools in Brandenburg, Germany. The schools were pre-selected to be representative in terms of urban and rural regions and socio-economic backgrounds. Trained and supervised experimenters assessed each child separately in a quiet room at home or school. All children completed the same test battery in two one-hour sessions approximately 1 week apart. The order of tests was block randomized (ABCD, BADC). Teachers and parents answered the questions online or on paper, and the class teachers received individual questionnaires for each participating child. For their participation, the children received vouchers (cinema, books) and gifts worth 20–30€. Additionally, for each child they provided information on, teachers received 5€ for the class fund. Each child's primary caregiver provided informed consent. The study was approved by the local Research Ethics Committee and the Ministry of Education, Youth, and Sport of the Federal State of Brandenburg.

### Participants

At T1, a total of 1654 children (52.2% female) aged 6 to 11 years (*M* = 8.36, *SD* = 0.95) and their parents (*N* = 1339) and teachers (*N* = 1421) were included in the study. Looking at the children’s age distribution, 9.1% were aged 6, 28.6% were aged 7, 33.0% were aged 8, 26.4% were aged 9, 2.7% were aged 10, and 0.2% were aged 11. At T2, 1609 children (51.9% female), now aged 7 to 11 years (*M* = 9.11, *SD* = 0.93), 1196 parents, and 1172 teachers participated again. At T3, the remaining sample consisted of 1501 children, now aged 9 to 13 years (51.7% female, *M* = 11.06 years, *SD* = 0.92 years), 1068 parents, and 1110 teachers. Concerning the highest reported educational level of the parents, 36.6% indicated a university degree, 15.5% high school diploma ("Abitur"), 25.5% higher secondary school, 2.1% lower secondary school, 0.9% special school or no degree and 19.6% did not provide information on their educational level. As a proxy for ethnic background, the language parents use to communicate with their child was used. 93.2% of the parents reported speaking German, 5.8% reported speaking German and at least one other language, and 1.0% reported not speaking German at home.

There was a low attrition rate for the children of 2.2% between T1 and T2 and 6.7% between T2 and T3. A binomial logistic regression was performed with the variables at T1 predicting participation at T2 and T3. Results indicated that participation at T2 (*b* = 1.78, *p* = 0.005) and T3 (*b* = 0.75, *p* = 0.035) was predicted by sex, with male participants being more likely to participate again. Additionally, participation at T2 was predicted by lower updating (*b* = −0.62,* p* = 0.020) and higher inhibition scores (*b* = 0.61, *p* = 0.005), and participation at T3 by lower parent-reported victimization (*b* = −0.72, *p* = 0.039), higher self-reported peer rejection (*b* = 1.33, *p* = 0.023), and higher inhibitory control (*b* = 0.40, *p* = 0.037); see supplementary materials (Table S2) for all model coefficients. As unit non-response depended on some of the observed variables, we cannot consider the data to be missing completely at random (Rubin, 1976). To account for missingness, we used the full information maximum likelihood estimation (FIML; Allison, 2001), which has shown unbiased results in the absence of data missing completely at random (Enders & Bandalos, 2001).

### Measures

#### Adverse Peer Experiences

APEs were assessed through parent-, teacher-, and self-reports at all three measurement points. Children rated two items of the social integration scale of a questionnaire measuring emotional and social school experiences (FEESS; Rauer & Schuck, 2003, 2004). Both items ("the other children often laugh at me", "I may join in playing in the schoolyard") were assessed on a dichotomous scale (*yes* (1), *no* (2)) for children in grades one and two. For children in higher grades, a 4-point scale (*yes, rather yes, rather no, no*) was applied, which was converted into a 1 (*yes, rather yes*) and 2 (*rather no, no*) scale for better comparability. Positively worded items were recoded. Sum scores across the two items were calculated and subtracted by one for each time point to heighten comparability to parent- and teacher-reported victimization given the same range of 1–3. We calculated ordinal alpha because conventional measures of scale reliability would have resulted in biased results due to the dichotomous scale (Gadermann et al., 2012). The sample indicated an ordinal alpha of α_T1_ = 0.72, α_T2_ = 0.63, and α_T3_ = 0.75.

Parents and teachers rated each child on the degree of perceived peer victimization in the last 6 months by each answering an item from the subscale Peer Relationship Problems from the Strength and Difficulties Questionnaire (SDQ; Goodman, 2001), namely "Picked on or bullied by other children." Respondents indicated on a three-point response scale whether the statement was *not true* (1), *somewhat true* (2), or *certainly true* (3). Combined parent- and teacher-report indicated an ordinal alpha of α_T1_ = 0.53, α_T2_ = 0.67, and α_T3_ = 0.68. Higher scores indicated more APEs for all items. Parent-, teacher-, and child-report were combined into the variable of APEs using latent modelling.

#### Updating

Updating was measured at all measurement points through the Digit Span Backwards Task of the German version of the Wechsler Intelligence Scale for Children (HAWIK-IV; Petermann & Petermann, 2008). Children were asked to repeat numerical sequences in reverse order. Each trial consisted of two sequences of the same length, which gradually increased in length between trials. The task ended after eight trials (16 sequences) or when both sequences were incorrectly repeated during one trial. The total number of correctly recalled numerical sequences determined the updating score.

#### Flexibility

Flexibility was measured at T1 and T2 through the computer-based Cognitive Flexibility Task (Röthlisberger et al., 2010; adapted from Zimmermann et al., 2002). Children were instructed to feed plain and multi-colored fish alternately, which were simultaneously presented on the left and right sides of the computer screen, with randomly changing sides across trials. The task consisted of 46 trials, separated by a break in which participants received positive feedback. As cognitive flexibility score, the number of correct responses in switch trials was used (trials that required a response pattern change from right-left to left-left or right-right).

At T3, the cognitive flexibility task was replaced by an adapted, computer-based version of the Dimensional Change Card Sorting task (Qu et al., 2013). Children were shown two symbols in different colors in the upper portion of the screen. They were instructed to sort another colored symbol in the lower part of the screen according to one of the two dimensions (color or symbol). The dimension to be sorted by was constant throughout the first block of 20 trials and altered in 12 out of 48 trials in the second block. The kids were told to press one of two keyboard keys as quickly and accurately as possible. The number of correct responses in switch trials was used as a flexibility score in the analysis.

#### Inhibition and Inhibitory Control

We distinguished between inhibition as an experimentally gathered state measure and inhibitory control, defined as the child's ability to suppress inappropriate behavior observed by their parents over an extended period.

##### Inhibition

Performance-based inhibition was measured at all measurement points through an adapted version of the fruit-vegetable Stroop task (Roebers et al., 2011). Each of the four trials consisted of one page depicting 25 stimuli (trial one colored rectangles; trial two correctly colored fruits and vegetables; trial three fruits and vegetables colored in grey; trial four incorrectly colored fruits and vegetables). The children were instructed to name the depicted color of the stimuli (trials 1 and 2) or the color the fruits and vegetables should have (trials 3 and 4) as fast as possible as the time per trial was measured. An interference score was calculated according to Röthlisberger et al. (2010) formula: Time trial 4 – ((time trial 1 × time trial 3) / (time trial 1 + time trial 3)). Scores were z-standardized and reversed so that higher scores indicated less interference.

##### Inhibitory Control

Inhibitory control was measured at all measurement points via parent-report through six items taken from the subscale inhibitory control of the Temperament in Middle Childhood Questionnaire (TMCQ; Simonds et al., 2007). The TMCQ is validated for children aged 7 to 11 but has been used for children up to the age of 13 (e.g., Affrunti et al., 2016). On a five-point scale ranging from *not true* (1) to *true* (5), parents rated their child's ability to suppress inappropriate responses in uncertain and new situations or under instruction in the last 6 months (e.g., "Can stop him/herself when s/he is told to stop"). Higher scores indicated higher inhibitory control. Mean scores across the six items were calculated for each time point. The scale showed an internal consistency of α_T1_ = 0.68, α_T2_ = 0.67, and α_T3_ = 0.71.

#### Emotional Reactivity

Emotional reactivity was measured at all measurement points via parent-report through ten items of the subscale emotion control taken from the Behavior Rating Inventory of Executive Function (BRIEF; Gioia et al., 2000). On a modified 5-point scale ranging from *never* (1) to *always* (5), parents rated children's ability to control their emotions in the last 6 months (e.g., "The mood is easily influenced by the situation"). The scaling was modified to avoid repeated changes in the answer format across questionnaires. It also allows a finer gradation. Higher scores indicated higher emotional reactivity. For each time point, mean scores were calculated. The samples' internal consistency was α_T1_ = 0.90, α_T2_ = 0.91, α_T3_ = 0.92.

#### Planning

Planning was measured at all measurement points through eight items in teacher-report on the subscale planning and organizing from the Behavior Rating Inventory of Executive Function (BRIEF; Gioia et al., 2000). On a modified 5-point scale ranging from *never* (1) to *always* (5), teachers rated children's ability to plan and organize things in advance in the last 6 months (e.g., "Does not plan tasks for school in advance"). In deviation from the original version of the BRIEF, items were reverse-scored so that higher scores indicated higher planning behavior. For each time point, mean scores across items were calculated. The scale showed an internal consistency of α_T1_ = 0.93, α_T2_ = 0.93, and α_T3_ = 0.95 in the sample.

### Data Analysis

Descriptive statistical analyses were performed using SPSS 29. M*plus* Version 7.3 (Muthén & Muthén, 2012) was used for structural equation modeling. For all analyses, the alpha level was set to *p* = 0.05 and FIML was used to account for missing data. For each of the three measurement points, APEs were modeled as a latent factor via confirmatory factor analysis, allowing the integration of ratings from different informants. The robust maximum likelihood estimator (MLR) accounted for deviations from normal distribution in all analyses. To test our hypotheses concerning a predictive bidirectional relationship of APEs and different SR facets during middle childhood, separate cross-lagged panel analyses were conducted for each SR facet. The SR facets were z-standardized and entered as manifest variables for time points T1–T3. Furthermore, to strengthen the validity of our cross-lagged panel models (CLPMs), we included a path from T1 to T3 (lag-2 effect) for the manifest SR facets. Lag-2 effects are discussed to help to control for confounding (Lüdtke & Robitzsch, 2022). We controlled for age and sex in all cross-lagged analyses. Established fit indices were used to assess the model fit with a good fit indicated by root mean square error of approximation (RMSEA) ≤ 0.05, comparative fit index (CFI) ≥ 0.95, and root mean square residual (SRMR) ≤ 0.10 (Beauducel & Wittmann, 2005). It is suggested that the χ^2^-value is highly inflated by many degrees of freedom and large sample sizes (Schermelleh-Engel et al., 2003). Therefore, we relied on the χ^2^ -value relative to its degrees of freedom. An indicator of a good model fit was a ratio under 2 (Tabachnick et al., 2007). Measurement invariance (MI) across time was assessed for APEs by comparing the constrained with a freed model through χ^2^-difference tests. As the MLR estimator does not allow for the conventional χ^2^ difference test, the adjusted procedure with Satorra-Bentler scale corrected χ^2^-values was employed. MI across time was not tested for the SR facets, as they were implemented as manifest variables. Effect sizes were considered as small 0.10–0.30, medium 0.30–0.50, and large 0.50–0.70 (Cohen, 1988). Regarding the interpretation of cross-lagged effects, effects of 0.03 were considered small, 0.07 medium, and 0.12 large (Orth et al., 2022).

Additionally, sex and age differences were explored through multigroup analyses. Therefore, separate structural equation models were estimated for female and male as well as younger and older children, respectively. Based on the age at T1, a median split (*Md* = 8.40 at T1) was used to divide the sample into a younger and an older sub-sample. MI across groups was assessed for each model for age and sex, respectively. The cross-lagged and autoregressive paths were then compared between groups using model constraints in M*plus*. Difference scores were calculated between groups and tested for significance.

## Results

### Descriptive Results and Confirmatory Factor Analysis

Bivariate correlations and descriptive measures of the parent-, teacher- and child-report of APEs at all three time points are summarized in Table 1. With percentages ranging from 78.3% in parent-reports at T1 to 87.9% in teacher-reports at T2, most children were reported not to be affected by APEs. Small to moderate correlations emerged between raters at the same and different time points and within raters at different time points. For T1 to T3, a multi-informant latent factor of APEs was specified, including parent-, teacher-, and self-reports as indicators. Metric MI across the three time points was given, as the model with the factor loadings constrained to be equal showed no significant difference to the baseline model (Δχ^2^ (4) = 7.54, *p* = 0.110). After allowing the intercepts to correlate within raters across time points (see supplementary material, Figure S1), the model indicated a good model fit (χ^2^ (19) = 21.60, χ^2^ / df = 1.14, RMSEA = 0.01, SRMR = 0.03, CFI = 1.0, *N* = 1657).

**Table 1:** Bivariate correlations between raters and time points, as well as descriptive measures of the parent-, teacher-, and child-reports of APEs at T1, T2, and T3

Variable	1	2	3	4	5	6	7	8	*N*	*M* (*SD*)	*Min* (%)	*Max* (%)
1. PR T1	—								1330	1.24 (0.48)	78.3	2.3
2. PR T2	0.46^***^	—							1185	1.21 (0.46)	81.0	2.0
3. PR T3	0.42^***^	0.41^***^	—						1058	1.22 (0.82)	80.5	2.9
4. TR T1	0.21^***^	0.22^***^	0.19^***^	—					1289	1.15 (0.37)	85.7	0.6
5. TR T2	0.25^***^	0.28^***^	0.32^***^	0.34^***^	—				1151	1.28 (0.35)	87.9	0.7
6. TR T3	0.22^***^	0.22^***^	0.33^***^	0.20^***^	0.24^***^	—			912	1.22 (0.49)	80.8	3.2
7. CR T1	0.23^***^	0.23^***^	0.22^***^	0.16^***^	0.20^***^	0.20^***^	—		1639	1.22 (0.50)	82.2	4.0
8. CR T2	0.16^***^	0.25^***^	0.20^***^	0.12^***^	0.17^***^	0.13^***^	0.31^***^	—	1583	1.20 (0.47)	82.8	3.0
9. CR T3	0.20^***^	0.20^***^	0.35^***^	0.14^***^	0.15^***^	0.28^***^	0.22^***^	0.23^***^	1499	1.16 (0.43)	86.8	2.6

*Min* (%) percent indicating minimum, *Max* (%) percent indicating maximum, *PR* parent-report, *TR* teacher-report, *CR* child-report, *T1* time 1, *T2* time 2, *T3* time 3

*p< 0.05; **p< 0.01; ***p< 0.001

Table 2 depicts descriptive measures of the SR facets updating, flexibility, inhibition, emotional reactivity, inhibitory control, and planning for T1–T3. Table 3 illustrates the bivariate correlations among all study variables, including the latent factor of APEs. Most SR facets indicated small to moderate positive correlations among each other. Small to moderate negative correlations were found between APEs and the SR facets except for APEs T1 with flexibility T3 and APEs T2 with flexibility and inhibition T3.

**Table 2:** Sample size and descriptive statistics of self-regulation facets for all three time points

		Updating	Flexibility	Inhibition	Emotional reactivity	Inhibitory control	Planning
T1	*N*	1638	1639	1641	1293	1310	1369
	*M* (*SD*)	6.18 (1.46)	15.40 (4.80)	24.92 (8.77)	2.20 (0.71)	3.53 (0.66)	3.71 (0.89)
	Range	0 – 13	0 – 22	—	1 – 5	1 – 5	1 – 5
	Study Range	0 – 13	0 – 22	7.07 – 89.03	1 – 4.50	1.17 – 5	1 – 5
T2	*N*	1605	1583	1602	1165	1173	1124
	*M* (*SD*)	6.63 (1.50)	18.15 (3.91)	20.53 (6.87)	2.13 (0.68)	3.59 (0.63)	3.64 (0.90)
	Range	0 – 13	0 – 22	—	1 – 5	1 – 5	1 – 5
	Study Range	0 – 13	0 – 22	5.91 – 66.41	1 – 4.60	1.33 – 5	1.13 – 5
T3	*N*	1493	1478	1487	1028	1053	1087
	*M* (*SD*)	7.38 (1.63)	9.77 (1.87)	16.71 (5.30)	2.24 (0.73)	3.75 (0.68)	3.68 (0.96)
	Range	0 – 13	0 – 12	—	1 – 5	1 – 5	1 – 5
	Study Range	0 – 13	0 – 12	1.75 – 52.34	1 – 4.80	1.33 – 5	1 – 5

*T1* time 1, *T2* time 2, *T3* time 3

**Table 3:** Bivariate correlations between study variables

Variable	1	2	3	4	5	6	7	8	9	10	11	12	13	14	15	16	17
1. T1 APEs	—																
2. T2 APEs	0.90^***^	—															
3. T3 APEs	0.75^***^	0.74^***^	—														
4. T1 Updating	−0.12^**^	−0.10^**^	−0.13^***^	—													
5. T2 Updating	−0.16^***^	−0.13^**^	−0.22^***^	0.45^***^	—												
6. T3 Updating	−0.14^***^	−0.13^**^	−0.12^***^	0.39^***^	0.45^***^	—											
7. T1 Flexibility	−0.25^***^	−0.19^***^	−0.21^***^	0.35^***^	0.33^***^	0.32^***^	—										
8. T2 Flexibility	−0.25^***^	−0.19^***^	−0.26^***^	0.31^***^	0.32^***^	0.27^***^	0.57^***^	—									
9. T3 Flexibility	−0.05	−0.03	−0.11^**^	0.10^***^	0.09^**^	0.14^***^	0.19^***^	0.22^***^	—								
10. T1 Inhibition	−0.21^***^	−0.13^**^	−0.16^***^	0.27^***^	0.25^***^	0.26^***^	0.34^***^	0.34^***^	0.16^***^	—							
11. T2 Inhibition	−0.20^***^	−0.13^**^	−0.20^***^	0.22^***^	0.27^***^	0.24^***^	0.33^***^	0.33^***^	0.17^***^	0.60^***^	—						
12. T3 Inhibition	−0.12^**^	−0.07	−0.10^**^	0.20^***^	0.21^***^	0.20^***^	0.28^***^	0.29^***^	0.16^***^	0.50^***^	0.53^***^	—					
13. T1 ER	0.40^***^	0.38^***^	0.25^***^	−0.09^**^	−0.10^***^	−0.05	−0.04	−0.06^*^	−0.06^*^	−0.09^**^	−0.12^***^	−0.05	—				
14. T2 ER	0.35^***^	0.41^***^	0.25^***^	−0.06^*^	−0.11^***^	−0.05	−0.05	−0.06	−0.02	−0.09^**^	−0.10^**^	−0.07^*^	0.71^***^	—			
15. T3 ER	0.35^***^	0.38^***^	0.35^***^	−0.05	−0.07^*^	−0.05	−0.01	−0.03	−0.03	−0.06	−0.09^**^	−0.04	0.63^***^	0.65^***^	—		
16. T1 IC	−0.38^***^	−0.35^***^	−0.23^***^	0.10^***^	0.13^***^	0.07^**^	0.14^***^	0.19^***^	0.09^**^	0.12^***^	0.11^***^	0.05	−0.41^***^	−0.34^***^	−0.34^***^	—	
17. T2 IC	−0.37^***^	−0.37^***^	−0.22^***^	0.06^*^	0.11^***^	0.05	0.15^***^	0.15^***^	0.10^**^	0.10^***^	0.11^***^	0.06^*^	−0.29^***^	−0.36^***^	−0.31^***^	0.66^***^	—
18. T3 IC	−0.31^***^	−0.31^***^	−0.22^***^	0.09^**^	0.13^***^	0.10^**^	0.15^***^	0.18^***^	0.12^***^	0.11^***^	0.11^***^	0.08^**^	−0.23^***^	−0.28^***^	−0.34^***^	0.58^***^	0.61^***^
19. T1 Planning	−0.56^***^	−0.46^***^	−0.39^***^	0.26^***^	0.27^***^	0.25^***^	0.30^***^	0.30^***^	0.18^***^	0.29^***^	0.28^***^	0.23^***^	−0.17^***^	−0.15^***^	−0.13^***^	0.30^***^	0.27^***^
20 T2 Planning	−0.46^***^	−0.46^***^	−0.40^***^	0.22^***^	0.27^***^	0.26^***^	0.28^***^	0.28^***^	0.16^***^	0.28^***^	0.26^***^	0.20^***^	−0.15^***^	−0.14^***^	−0.15^***^	0.27^***^	0.26^***^
21 T3 Planning	−0.44^***^	−0.43^***^	−0.41^***^	0.17^***^	0.17^***^	0.16^***^	0.21^***^	0.21^***^	0.14^***^	0.21^***^	0.18^***^	0.17^***^	−0.20^***^	−0.20^***^	−0.20^***^	0.26^***^	0.24^***^
22. Sex	0.03	0.07	0.01	0.02	−0.01	0.00	−0.15^***^	−0.09^***^	−0.06^*^	−0.08^**^	−0.09^***^	−0.07^**^	−0.01	−0.03	−0.05	−0.13^***^	−0.14^***^
23. Age	0.08^*^	0.08	0.00	0.25^***^	0.19^***^	0.10^***^	0.29^***^	0.22^***^	0.05	0.35^***^	0.32^***^	0.24^***^	0.03	0.02	−0.01	0.04	0.04

Variable	18	19	20	21	22	23											
18. T3 IC	—																
19. T1 Planning	0.30^***^	—															
20 T2 Planning	0.30^***^	0.81^***^	—														
21 T3 Planning	0.29^***^	0.58^***^	0.63^***^	—													
22. Sex	−0.10^**^	−0.21	−0.25	−0.19	—												
23. Age	0.04	0.00	−0.01	−0.09^**^	0.03	—											

*APEs* adverse peer experiences, *ER* emotional reactivity, *IC* inhibitory control, *T1* time 1, *T2* time 2, *T3* time 3, Sex: 1 = female, 2 = male

*p< 0.05; **p< 0.01; ***p< 0.0001

### Hypotheses Testing

**Fig. 1:**
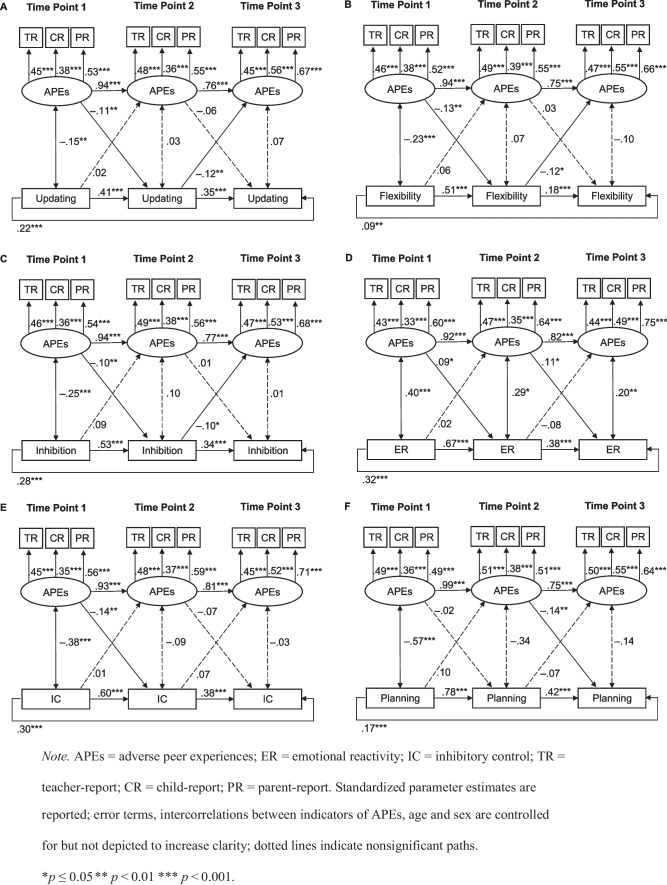
Cross-lagged models of APEs and the different SR facets

To test the hypotheses of a negative bidirectional relation between APEs and SR, we next modeled six CLPMs – one for each SR facet. All models indicated a good model fit, as presented in Table 4. The results for each CLPM and the factor loadings for APEs are presented in Fig. 1. As depicted, APEs indicated large stabilities over time. Most SR facets showed medium to large stabilities over time, with the exception of flexibility T2 to T3 and most paths from T1 to T3, which indicated small stabilities over time. Concerning the prediction of SR by APEs, APEs T1 predicted emotional reactivity T2 (β = 0.09, *SE* = 0.04, *p* = 0.033), inhibitory control T2 (β = –0.14, *SE* = 0.04, *p* = 0.001), flexibility T2 (β = –0.13, *SE* = 0.04, *p* = 0.002), updating T2 (β = –0.11, *SE* = 0.04, *p* = 0.002) and inhibition T2 (β = –0.10, *SE* = 0.04, *p* = 0.004). Since higher values of emotional reactivity reflect lower SR, a negative prediction of APEs T1 is shown for all significant SR facets at T2. APEs T2 predicted emotional reactivity T3 (β = 0.11, *SE* = 0.04, *p* = 0.013) and planning T3 (β = –0.14, *SE* = 0.04, *p* = 0.001). No significant paths emerged between the SR facets at T1 and APEs at T2. Regarding the prediction of SR T2 to APEs T3, flexibility T2 (β = –0.12, *SE* = 0.05, *p* = 0.024), updating T2 (β = –0.12, *SE* = 0.04, *p* = 0.005) and inhibition T2 (β = –0.10, *SE* = 0.05, *p* = 0.023) predicted APEs at T3. The reported significant paths imply medium to large effects (Orth et al., 2022).

**Table 4:** Model fit indices for cross-lagged analyses of APEs and the different SR facets

Model	χ^2^	df	*p*	χ^2^ / df	RMSEA (90% CI)	SRMR	CFI
Updating	91.55	52	0.001	1.76	0.021 (0.014; 0.029)	0.031	0.979
Flexibility	76.17	52	0.016	1.47	0.017 (0.007; 0.024)	0.029	0.986
Inhibition	80.81	52	0.006	1.55	0.018 (0.010; 0.026)	0.029	0.987
Emotional reactivity	83.95	52	0.003	1.61	0.019 (0.011; 0.027)	0.031	0.985
Inhibitory control	70.24	52	0.047	1.35	0.015 (0.002; 0.023)	0.027	0.991
Planning	101.76	52	< 0.001	1.96	0.024 (0.017; 0.031)	0.029	0.981

*CFI* comparative fit index, *RMSEA* root mean square error of approximation, *SRMR* standardized root mean square residual; control variables were sex and age at time 1

### Sex and Age Differences

Sex and age differences across time were explored through multigroup analyses. Scalar MI was given for multigroup analysis across sex, and partial scalar MI for multigroup analysis across age (see supplementary material Table S3). Separate models were estimated for female and male and younger and older children, respectively. All models indicated good model fits (see supplementary material, Table S4). Paths were then compared between groups. All significant age and sex differences for stabilities and cross-lagged paths are presented in Table 5. Looking at the cross-lagged paths, a significant sex difference was found for APEs T1 and flexibility T2 (*Δb* = 0.20, *p* = 0.019). While for female participants experiencing more APEs at T1 predicted lower flexibility scores (β = −0.22, *p* = < 0.001), no significant prediction was found for male participants (β = –0.04, *p* = 0.531). Additionally, the model of flexibility and APEs indicated an age difference for the prediction of APEs T3 from flexibility T2 (*Δb* = −0.46, *p* = 0.011), which was suggested for younger (β = –0.26, *p* = 0.001), but not for older children (β = 0.03, *p* = 0.688). None of the other cross-lagged paths indicated significant age or sex differences.

**Table 5:** Results of multigroup analyses of age and sex

Model	*Δb*	*SE*	*p*	β	*SE*	*p*	β	*SE*	*p*
Age	Difference			Older children			Younger children		
Inhibition T1 → inhibition T3	−0.20	0.07	0.008	0.36	0.04	< 0.001	0.23	0.05	< 0.001
Sex → inhibition T1	−0.23	0.09	0.017	−0.04	0.04	0.260	−0.13	0.03	< 0.001
Flexibility T2 → APEs T3	−0.46	0.18	0.011	0.03	0.07	0.688	−0.26	0.07	< 0.001

Sex	Difference			Male			Female		
APEs T1 → flexibility T2	0.20	0.09	0.019	−0.04	0.06	0.531	−0.24	0.06	< 0.001
Age → flexibility T2	−0.11	0.05	0.033	0.03	0.04	0.419	0.13	0.03	< 0.001
Inhibition T1 → inhibition T3	−0.19	0.08	0.011	0.21	0.05	< 0.001	0.36	0.05	< 0.001
Age → inhibition T1	0.11	0.05	0.038	0.38	0.03	< 0.001	0.32	0.03	< 0.001
Updating T1 → updating T3	−0.11	0.05	0.034	0.16	0.03	< 0.001	0.27	0.04	< 0.001

Age by median split at T1 (*Md* = 8.40)

*APEs* adverse peer experiences, *T1* time 1, *T2* time 2, *T3* time 3; Sex: 1 = female, 2 = male

## Discussion

Since peer experiences and SR skills are considered crucial for healthy child development (Poulin & Chan, 2010), the current prospective study investigated the longitudinal associations of APEs and the six SR facets updating, flexibility, inhibition, emotional reactivity, inhibitory control, and planning. We found partial confirmation of our hypotheses proposing a bidirectional negative prospective association between APEs and SR: Experiencing more APEs at T1 predicted lower performance of the three basal SR facets updating, inhibition, and flexibility at T2, which, in turn, predicted more APEs at T3. Additionally, APEs at earlier measurement points predicted higher emotional reactivity at T2 and T3, lower inhibitory control at T2, and lower planning at T3.

### Prediction of SR by APEs

Regarding the prediction of SR by APEs, we observed a far-reaching link. Our results show that APEs at T1 predicted reduced SR capacities at T2 for all basal SR facets (emotional reactivity, inhibition, inhibitory control, updating, and flexibility). The number of SR facets predicted by APEs decreased over time, with APEs at T2 predicting lower planning behavior and higher emotional reactivity at T3. All these significant effects can be considered as medium to large effects (Orth et al., 2022). Our results indicate the importance of APEs in predicting SR facets in middle childhood. A possible explanation for this association could be that rejected and victimized children experience few reciprocal friendships and relative isolation in classroom social networks (Boivin et al., 1995). This isolation could leave them with fewer opportunities to practice SR skills in friendships and in class, leading to fewer SR capabilities compared to non-victimized and rejected children their age. Additionally, our findings confirm that SR can be predicted by negative life experiences (Hughes, 2011).

The results extend previous research by examining different SR facets separately. The basal SR facets inhibition, inhibitory control, updating, and flexibility at T3 were not predicted by APEs at T2, which may be explained by a distinct temporal development of basal vs. complex SR facets. A meta-analysis by Romine and Reynolds (2005) indicated an apparent increase in inhibition and flexibility performance until the age of 11, with only marginal improvements between 11 and 14 years and no performance increase thereafter. This increasing consolidation might make the basal facets less susceptible to external life events. Unlike the basal facets, the complex facet of planning has shown age-related improvements throughout childhood and adolescence (Romine & Reynolds, 2005) and is expected to be especially relevant for older children and adolescents (Bailey & Jones, 2019). Our results align with these findings, as we observed a prediction of planning by APEs to occur only later in middle childhood.

Of note, APEs predicted heightened emotional reactivity throughout the study – demonstrating a persistent negative prediction of earlier APEs on later emotional SR. This is consistent with prior research showing that adolescents who experience APEs are more likely to have emotion regulation difficulties (for a review, see Herd & Kim-Spoon, 2021). Overall, the distinct results with respect to the different SR facets illustrate the importance of investigating SR facets separately rather than examining a global score.

### Prediction of APEs by SR

Contrary to our previously formulated hypothesis, no significant paths were found from SR at T1 to APEs around 9 months later at T2. These results are partly in contrast (Holmes et al., 2016; Stenseng et al., 2015) and partly in agreement with previous research (de Wilde et al., 2016; Lecce et al., 2020). A different pattern of results was found for the second and third measurement points, with the children now being older on average. Lower updating, flexibility, and inhibition at T2 predicted more APEs at T3. Large effect sizes emerged for the prediction of APEs at T3 by updating at T2 and flexibility at T2, and medium effect sizes were found for the prediction of APEs at T3 by inhibition at T2.

These results align with studies indicating a predictive association between EF and later peer problems in middle childhood (e.g., Holmes et al., 2016). EF is associated with social adjustment, social competence (McKown et al., 2009), and the ability to resolve conflicts (David & Murphy, 2007). Therefore, it seems reasonable to assume that children with higher basal SR skills are able to make friends and resolve conflicts, while the opposite might be true for children with lower basal SR skills. The higher stability of basal SR facets with increasing age (Romine & Reynolds, 2005) might make differences between children more prone and salient in comparisons over time. This heightened visibility could lead to children with lower skills becoming easier targets for APEs. Therefore, the predictive role of SR for social outcomes might emerge later in a child’s school career. It is also possible that the predictive association of SR on APEs only becomes apparent over a longer time period. In the German school system, students spend nearly all classes with the same other students. Children within one group might need time to form impressions regarding their peers’ SR before the mere presence of low SR predicts APEs which might not have been possible within the 9 months. However, the observed predictions of APEs by inhibition, updating, and flexibility over the 2-year period between T2 and T3 underline the relevance of the associations. Generally, middle childhood is characterized by many developments and changes in self-regulation (Romine & Reynolds, 2005) and APEs (Hong & Espelage, 2012), making it essential to consider interindividual differences within the period.

Interestingly, the three facets updating, flexibility, and inhibition, which predicted APEs at T3, were all task-based measures of SR. Research has indicated that task-based measures and ratings of EF show no or only small correlations, leading to the assumption that they might assess different constructs (Friedman & Gustavson, 2022; Toplak et al., 2013). In the current study, inhibition (task-based) and inhibitory control (rating) indicated only small correlations, despite both measures intended to measure a related construct. The low correlation could be explained by the different time perspectives of the measures, as inhibition measured the current performance, whereas the rating of inhibitory control referred to the last 6 months. The underlying processes and demands on inhibition may differ between tasks that assess inhibition in a few milliseconds with clearly identifiable goals and parent-reports assessing typical child behavior over 6-months in concrete situations (Friedman & Gustavson, 2022). Our results suggest that child performance is more relevant for predicting APEs by SR than the average inhibitory control reported in the last few months. In addition, the data stem from different sources—as the children completed the tasks, while the ratings were provided by parents. The informants differ in their perspective (self vs. other) and the context (universal vs. home), which may explain the lack of prediction from parent-reported inhibitory control T2 to APE T3 (Makol et al., 2020). Parents’ report can only refer to observed child behavior in situations they experience together, which may differ from children’s behavior at school and with peers. Therefore, parents may have limited insight, especially as children spend more unsupervised time with their peers as they get older (Lam et al., 2014). In summary, the differences in the results on inhibition (task-based) and inhibitory control (rating) support the notion that slightly different constructs are measured by tasks and ratings.

Taken together, as hypothesized, a proposed bidirectional relationship was observed for the three basal SR facets updating, flexibility, and inhibition. Following a mutually reinforcing downward spiral model (Slater et al., 2003), more victimization and social rejection preceded lower updating, flexibility, and inhibition, which in turn predicted more APEs. To illustrate, a child experiencing peer victimization and rejection at T1 has more difficulty than children without such experiences in suppressing unwanted impulses, switching between different strategies, and keeping information active in working memory. A child having lower scores in these SR facets then in turn experiences more adverse experiences by peers compared to other children. This outlined process may contribute as a vicious circle to the stability of victimization and peer rejection found in our study and previous research (Pouwels et al., 2016). The relevance of this vicious circle is underlined by the medium to large effect sizes found.

Our results highlight the importance of early APEs prevention programs. Anti-bullying programs have been found to be more effective when implemented before the age of 10 (Jiménez-Barbero et al., 2016). Therefore, and in line with our observations, we recommend their implementation in early school years to prevent long-lasting APEs and problems in SR development. Of note, contrary to Holmes et al. (2016), no spiral model from low SR abilities over more APEs to lower SR capabilities was found in middle childhood. It should be noted, however, that the time interval and age in the study conducted by Holmes et al. (2016), with a mean age of 4.5 years at T1 and 9/10 years at T2, differed from our study. It is therefore possible that the first time interval in our study was too short or that middle childhood is not the critical age for demonstrating a spiral model starting from low SR via more APEs to lower SR.

### Sex and Age Differences

Sex and age differences in the reciprocal relations between APEs and SR were exploratively analyzed. Concerning sex differences, APEs at T1 predicted lower flexibility scores for female but not for male children. None of the other predictive associations between APEs and the different SR facets indicated sex differences. Previous studies showed an inconsistent picture, as they indicated both no sex differences (Holmes et al., 2016; Lecce et al., 2020; Stenseng et al., 2015) or sex-specific effects (Hawes et al., 2012; Jenkins et al., 2018). Further research focusing on possible sex and gender differences is warranted. Turning to age effects, the only significant age difference emerged for flexibility at T2 predicting APEs at T3, which was indicated for younger but not older children. The used Cognitive Flexibility Task (Röthlisberger et al., 2010; adapted from Zimmermann et al., 2002) was originally adapted for children up to the age of 7. The age effect may therefore indicate that at T2, the instrument was not appropriate for older children and could not differentiate between participants.

### Strengths, Limitations, and Future Implications

The present study provides initial data on the prospective relation of APEs and six different SR facets. Strengths of the study include the multi-method, multi-informant design and the inclusion of three measurement points spanning middle childhood. An additional strength is the large community sample, which had a low drop-out rate and included an equal number of male and female participants, allowing for comparisons across sex.

Nevertheless, some limitations should be mentioned. First, several limitations relate to the variable of APEs. Due to data privacy concerns, no investigation of APEs via peer report was possible. Including peer reports would be beneficial, as subjectivity is reduced due to multiple peers functioning as raters. Therefore, research proposes to combine peer- and self-report whenever possible (Volk et al., 2017). Not all of the interval between measurement points was covered since APEs were assessed during the previous 6 months. APEs that occurred beyond the previous 6 months remained unobserved. Additionally, the low reliability of the combined parent- and teacher-report in our study should be mentioned. This could be explained by the different contexts (home vs. school) in which the two informants see the child (De Los Reyes & Makol, 2021). However, the combination of parent-, teacher-, and self-reports allowed us to use latent variable modeling to investigate APEs and increased the ecological validity of our results. Second, SR facets were entered into the model as manifest variables, which did not allow testing for MI across time. Future studies should assess SR facets with multiple indicators to apply latent variable modeling. However, CLPMs can be conducted with both manifest and latent variables without showing a difference in the size of the effects (Orth et al., 2022). Third, it should be noted that an age-appropriate task adjustment required a change in the instrument used to measure flexibility at T3. However, both instruments are well-established experiments (Qu et al., 2013; Roebers et al., 2011). Fourth, the different mean time intervals between T1 to T2 and T2 to T3 need to be considered. Age and time intervals between measurement points increased, resulting in the assessment of prospective associations for unequal periods. Given the wide age range (6 to 11 years at T1), with some age groups being represented at T1 and T3, no specific conclusions about age-related patterns can be drawn.

A final limitation relates to the CLPM. There is an ongoing debate about the causal interpretability of CLPM as well as the drawbacks of this approach (e.g., Berry & Willoughby, 2017; Lüdtke & Robitzsch, 2022). The CLPM has been criticized for not allowing a distinction between within-person and between-person variance (e.g., Lucas, 2023; Usami et al., 2019). The random intercept cross-lagged panel model (RI-CLPM; Hamaker et al., 2015) is discussed as a possible alternative, as the development within and between children can be examined. However, CLPMs have been shown to be more consistent across different samples than the RI-CLPM (Orth, 2021), and the RI-CLPM requires at least four measurement time points to control for the influence of measurement errors (Lüdtke & Robitzsch, 2022; Usami et al., 2019). The RI-CLPM assesses temporal variation around the individual mean rather than the consequences of processes between persons, making it difficult to test causal hypotheses (Lüdtke & Robitzsch, 2022; Orth, 2021). Given our interest in potential rank order differences—e.g., whether children with low SR skills are at higher risk of experiencing APEs than children with high SR skills—we decided to use the CLPM. However, given the ongoing debate about the causal interpretability of the CLPM and the observational nature of the data, we cannot make any causal statements.

The current study indicated associations between APEs and distinct SR facets in six separate CLPMs. Our results highlight the importance of differentiating between SR facets as different patterns of results emerged. Future studies integrating multiple SR facets simultaneously into one model are needed to take the interplay between different SR facets into account and identify the most crucial facets. It is important to note that differences between types of APEs (peer victimization vs. peer rejection) were not considered in the present study. We encourage future research to differentiate between forms of APEs to understand their unique association with different SR facets. Moreover, we strongly recommend future research to examine individual temporal differences, for example, by employing the RI-CLPM or the autoregressive latent trajectory model (ALT; Bollen & Curran, 2004). Studies using these models are needed to extend our knowledge of developmental differences in children. This is especially important as different results were found depending on the method used (e.g., Littlefield et al., 2022; Orth, 2021).

## Conclusion

We tested the prospective relations between APEs (peer victimization and peer rejection) and six SR facets in separate CLPMs across three measurement points. Our results indicated a broad prediction of SR by APEs at the study's onset and a bidirectional relationship of updating, flexibility, and inhibition with APEs. Precisely, we observed that higher levels of APEs predicted lower levels of SR abilities, which in turn predicted higher APEs. Additionally, APEs predicted poorer SR abilities across all facets examined in this study, highlighting the far-reaching association of victimization and rejection on children's SR development. Notably, the different SR facets were differentially predicted at different time points during middle childhood, illustrating the importance of investigating SR facets separately rather than as a global score.

### Supplementary Information

Below is the link to the electronic supplementary material.

Supplementary file1 (DOCX 56 KB)

## Data Availability

The datasets generated and analyzed during the current study are not publicly available, as the participants were not asked to consent to publication within repositories but are available from the corresponding author upon reasonable request.
