# Supplementary material for: The Longitudinal Interplay between Adverse Peer Experiences and Self-Regulation Facets: A Prospective Analysis during Middle Childhood
Source: Res Child Adolesc Psychopathol. 2023 Oct 2;52(2):293–308. doi: 10.1007/s10802-023-01117-1 (PMC10937773; doi:10.1007/s10802-023-01117-1)
Supplement: Supplementary file 1 — Supplementary file1 (DOCX 56 KB) [file 10802_2023_1117_MOESM1_ESM.docx]

**SUPPLEMENTARY MATERIALS**

**Figure S1**

*Indicators, factor loadings, and intercorrelations of the latent variable measuring adverse peer experiences*

**
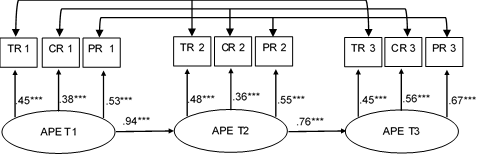
**

*Note.* APEs = adverse peer experiences; TR = teacher report; CR = child report; PR = parent report (PR); T1 = time point 1; T2 = time point 2; T3 = time point 3.

**p* ≤ .05 ** p < .01 *** p < .001.

**Table S1**

| **Deviation** | **Reason** |
| --- | --- |
| Naming of the SR facet *behavioral control* was changed to *inhibitory control* | To ensure unified naming within the research group. Additionally, inhibitory control corresponds to the name of the subscale of the Temperament in Middle Childhood Questionnaire (TMCQ; Simonds et al., 2007) and expresses the belonging to *inhibition* more clearly. |
| Use of the SR facet *emotional reactivity* in exchange for preregistered *emotion regulation* | Reliabilities for the emotion regulation items of the scale 'dealing with rage' of the questionnaire for measuring emotion regulation of children and adolescents (Fragebogen zur Erhebung der Emotionsregulation bei Kindern und Jugendlichen (FEEL-KJ); Grob & Smolenski, 2009) were not acceptable in our sample (α_T1_ = .31, α_T2_ = .23 α_T3_ =.35). Creating a sum score across all emotion regulation strategies was therefore not adequate. Also, an adapted approach, according to Krahé and Rohlf (2015), was not feasible due to fewer items available at T3. Consequently, we decided to forgo this questionnaire and investigate and report the related construct of emotional reactivity. |

*Deviations from preregistration*

*Note.* Preregistration is available at <https://osf.io/r6t3s>.

**Table S2**

*Results of the binomial logistic regressions predicting participation at T2 and T3*

|  | *b* | *SE* | Wald | *p* | Odds Ratio | 95% CI for Odds Ratio |
| --- | --- | --- | --- | --- | --- | --- |
| Participation T2 |  |  |  |  |  |  |
| Age | 0.222 | 0.297 | 0.560 | .454 | 1.249 | 0.698 / 2.234 |
| Sex | 1.780 | 0.632 | 7.930 | .**005** | 5.928 | 1.718 / 20.460 |
| APEs PR | 0.42 | 0.553 | 0.006 | .939 | 1.043 | 0.353 / 3.085 |
| APEs TR | −0.864 | 0.556 | 2.413 | .120 | 0.422 | 0.142 / 1.254 |
| APEs CR | 0.354 | 0.551 | 0.412 | .521 | 1.424 | 0.484 / 4.193 |
| Updating | −0.617 | 0.264 | 5.439 | **.020** | 0.540 | 0.321 / 0.906 |
| Flexibility | 0.381 | 0.246 | 2.392 | .122 | 1.463 | 0.903 / 2.371 |
| Inhibition | 0.612 | 0.216 | 7.991 | .**005** | 1.844 | 1.206 / 2.818 |
| Emotional reactivity | −0.276 | 0.280 | 0.973 | .324 | 0.758 | 0.438 / 1.314 |
| Inhibitory control | 0.113 | 0.276 | 0.169 | .681 | 1.120 | 0.652 / 1.924 |
| Planning | −0.079 | 0.316 | 0.062 | .803 | 0.924 | 0.498 / 1.716 |
| Constant | 0.171 | 2.845 | 0.004 | .952 | 1.186 |  |
| Participation T3 |  |  |  |  |  |  |
| Age | −0.155 | 0.190 | 0.667 | .414 | 0.856 | 0.590 / 1.242 |
| Sex | 0.745 | 0.353 | 4.458 | **.035** | 2.106 | 1.055 / 4.206 |
| APEs PR | −0.138 | 0.331 | 0.174 | .677 | 0.871 | 0.456 / 1.665 |
| APEs TR | −0.715 | 0.347 | 4.242 | **.039** | 0.489 | 0.248 / 0.966 |
| APEs CR | 1.334 | 0.585 | 5.199 | **.023** | 3.797 | 1.206/ 11.952 |
| Updating | −0.148 | 0.187 | 0.629 | .428 | 0.862 | 0.597 / 1.244 |
| Flexibility | 0.198 | 0.174 | 1.293 | .255 | 1.219 | 0.867 / 1.714 |
| Inhibition | −0.27 | 0.197 | 0.019 | .891 | 0.973 | 0.662 / 1.431 |
| Emotional reactivity | −0.044 | 0.175 | 0.062 | .803 | 0.957 | 0.680 / 1.349 |
| Inhibitory control | 0.396 | 0.190 | 4.346 | **.037** | 1.486 | 1.024 / 2.155 |
| Planning | 0.328 | 0.195 | 2.819 | .093 | 1.389 | 0.947 / 2.037 |
| Constant | 1.572 | 2.024 | 0.603 | .437 | 4.816 |  |

*Note.* Degrees of freedom were 1 for all Wald statistics. APEs = adverse peer experiences; PR = parent-report; TR = teacher-report; CR = child-report; T1 = time 1; T2 = time 2; T3 = time 3; The model was statistically significant for participation at T2 (χ²(11) = 24.155, *p* = .012) and T3 (χ²(11) = 28.094, *p* = .003). Significant results are printed in bold.

**Table S3**

*Model fits of baseline, metric, and scalar models for multigroup analyses of age and gender*

| Model | χ2 | df | p | χ2/df | RMSEA (90% CI) | CFI | SRMR | Scaling H0 |
| --- | --- | --- | --- | --- | --- | --- | --- | --- |
| **Age** |  |  |  |  |  |  |  |  |
| Baseline | 25.915 | 30 | .679 | 0.86 | <0.001 (0.000-0.026) | 1.00 | 0.021 | 2.021 |
| Metric | 34.831 | 39 | .661 | 0.89 | <0.001 (0.000-0.020) | 1.00 | 0.038 | 2.028 |
| Scalar | 44.779 | 43 | .397 | 1.18 | 0.007 (0.000-0.025) | 0.99 | 0.039 | 2.092 |
| **Gender** |  |  |  |  |  |  |  |  |
| Baseline | 22.698 | 30 | .8275 | 0.76 | <0.001 (0.000-0.016) | 1.00 | 0.020 | 2.005 |
| Metric | 28.795 | 39 | .8845 | 0.74 | <0.001 (0.000-0.020) | 1.00 | 0.028 | 2.011 |
| Scalar | 31.772 | 45 | .932 | 0.71 | <0.001 (0.000-0.007) | 1.00 | 0.027 | 2.107 |

# *Note.* CFI = comparative fit index; RMSEA = root mean square error of approximation; SRMR = standardized root mean square residual; Gender: 1 = female, 2 = male*;* age by median split at T1 (*Md* = 8.40). Satorra-Bentler scaled χ^2^-difference tests were used to compare models.

**Table S4**

*Model fits of each cross-lagged panel model investigating age and gender differences in the prospective relation of adverse peer experiences and the SR facets*

| Model | χ2 | df | *p* | χ2 / df | RMSEA (90% CI) | SRMR | CFI |
| --- | --- | --- | --- | --- | --- | --- | --- |
| **Age** |  |  |  |  |  |  |  |
| Updating | 111.26 | 97 | .153 | 1.15 | 0.013 (0.000; 0.024) | 0.033 | 0.991 |
| Flexibility | 90.40 | 97 | .670 | 0.93 | 0.000 (0.000; 0.015) | 0.030 | 1.00 |
| Inhibition | 112.73 | 97 | .131 | 1.16 | 0.014 (0.010; 0.024) | 0.030 | 0.991 |
| ER | 98.38 | 97 | .442 | 1.01 | 0.004 (0.000; 0.019) | 0.032 | 0.999 |
| IC | 84.55 | 97 | .813 | 0.87 | 0.000 (0.000; 0.012) | 0.029 | 1.00 |
| Planning | 118.27 | 97 | .070 | 1.22 | 0.016 (0.000; 0.026) | 0.032 | 0.992 |
| **Gender** |  |  |  |  |  |  |  |
| Updating | 135.40 | 99 | .009 | 1.37 | 0.021 (0.011; 0.029) | 0.037 | 0.980 |
| Flexibility | 121.61 | 99 | .061 | 1.23 | 0.017 (0.000; 0.026) | 0.036 | 0.987 |
| Inhibition | 127.37 | 99 | .029 | 1.29 | 0.019 (0.006; 0.027) | 0.034 | 0.987 |
| ER | 123.15 | 99 | .051 | 1.24 | 0.017 (0.000; 0.026) | 0.037 | 0.989 |
| IC | 120.68 | 99 | .068 | 1.22 | 0.016 (0.000; 0.026) | 0.034 | 0.989 |
| Planning | 143.05 | 99 | .003 | 1.45 | 0.023 (0.014; 0.031) | 0.035 | 0.982 |

*Note*. CFI = comparative fit index; RMSEA = root mean square error of approximation; SRMR = standardized root mean square residual; ER = emotional reactivity; IC = inhibitory control.
